# Epstein-Barr virus encoded latent membrane protein 1 regulates mTOR signaling pathway genes which predict poor prognosis of nasopharyngeal carcinoma

**DOI:** 10.1186/1479-5876-8-30

**Published:** 2010-03-26

**Authors:** Jing Chen, Chun-Fang Hu, Jing-Hui Hou, Qiong Shao, Li-Xu Yan, Xiao-Feng Zhu, Yi-Xin Zeng, Jian-Yong Shao

**Affiliations:** 1 State Key Laboratory of Oncology in Southern China, Sun Yat-Sen University Cancer Center, Guangzhou, China; 2 Department of Pathology, Sun Yat-Sen University Cancer Center, Guangzhou, China; 3 Department of Microbiolgy, Tumor and Cell Biology, Karolinska Institutet, Box 280, Stockholm SE-17177, Sweden; 4 Institute of Cancer Studies, University of Birmingham, Birmingham, UK, B15 2TT, UK; 5 Department of Experiment Research, Sun Yat-Sen University Cancer Center, Guangzhou, China

## Abstract

**Background:**

The oncoprotein Epstain-Barr Virus (EBV)-encoded latent membrane protein1 (LMP1) modulates the pathological effects of the NF-κB, AP-1 and JAK/STAT pathways in nasopharyngeal carcinoma (NPC).

**Methods:**

Microarray analysis was performed on the NPC cell line HONE1 stably transfected with a LMP1-expression plasmid or an empty vector. Based on assigned pathways analyzed using the KEGG database, the mTOR signaling pathway was selected for verification by quantitative RT-PCR. Western blot, RNA interference and immunofluorescence were used to determine the relationship between LMP1 and mTOR signing pathway genes, and their clinical significance to NPC.

**Results:**

Our studies revealed that overexpression of LMP1 upregulated the mTOR signaling pathway, possibly through phosphorylation of AKT/mTOR/P70S6K/4EBP1 in the NPC cell lines HONE1 and 6-10B. Knockdown of LMP1 reduced expression of p-mTOR and p-4EBP1 in EBV-positive NPC cell line C666-1. In addition, LMP1 expression closely correlated with expression of p-mTOR, p-P70S6K and p-4EBP1 in NPC tumors. Expression of p-P70S6K, p-4EBP1 and LMP1, but not p-mTOR, significantly correlated with overall survival of NPC patients. However, only LMP1 was an independent prognostic factor.

**Conclusions:**

These results suggest that the mTOR signaling pathway is regulated by LMP1 expression in NPC. LMP1 and the genes in the mTOR pathway such as p-P70S6K and p-4EBP1 may be potential prognostic biomarkers.

## Background

Nasopharyngeal carcinoma (NPC) is a unique cancer of the head and neck that has a high incidence in Southern China, where it is endemic, at 25 cases per 100,000 person-years in the Guangzhou area [1]. Most NPC patients can be cured if the disease is diagnosed and treated at an early stage. However, the long-term survival rate of NPC patients with advanced stage cancer is still very poor, with a median survival time for patients with distant metastasis of only 9 months [2].

Epstein-Barr virus (EBV) is a human herpesvirus that has been intimately associated with both lymphoid and epithelial malignancies including lymphoma, NPC and gastric cancer [3]. NPC tumor cells express a limited set of EBV latent genes including EBV nuclear antigen 1, latent membrane proteins (LMP1, LMP2A, LMP2B), and EBV-encoded small RNA [4]. Of these genes, LMP1 has been identified as encoding an oncoprotein that is thought to be a key modulator in NPC pathogenesis. In NPC, LMP1 contributes to invasion and metastasis by inducing expression of matrix metalloproteinase 9 (MMP9)[5]. In addition, LMP1 may mediate various pathological effects such as promotion of cell proliferation, metastasis and inhibition of apoptosis in NPC [6]. As a member of the tumor necrosis factor receptor superfamily, LMP1 expression can activate the nuclear factor-kappa B (NF-κB), activator protein 1 (AP-1) and employing Janus kinases (JAKs) or and signal transducers and activators of transcription (STATs) (JAK/STAT) pathways and regulate their substrates[6]. LMP1 also targets the phosphatidylinositol-3-kinase (PI3K)/AKT pathway to induce fibroblast transformation and enhance cell survival [7,8]. Moreover, LMP1 can promote epithelial cell motility and enhance invasiveness by activating the extracelluar signal-regulated kinase/mitogen-activated protein kinase (ERK-MAPK) pathway [9].

Mammalian target of rapamacin (mTOR) is an evolutionarily conserved serine/threonine protein kinase with an important role in cell growth and proliferation through regulation of ribosome biogenesis and protein translation [10]. PI3K/AKT is considered a critical upstream mediator of the mTOR signaling pathway. The characterized downstream effectors of mTOR are ribosomal protein S6 kinases (P70S6K), and eukaryotic initiation factor 4E (eIF4E)-binding protein (4E-BP1), with eIF4E dissociating from 4E-BP1 to initiate translation after 4E-BP1 phosphaorylation, while P70S6K translates mRNA transcripts with a 5'-TOP motif following hyperphosphorylation by mTOR [11,12].

To further clarify the signaling pathways regulated by LMP1 in NPC, we investigated the association between the mTOR signaling pathway and LMP1, the expression of p-mTOR, p-P70S6K and p-4EBP1, and their relationship to clinicopathologic parameters of NPC patients.

## Materials and methods

### Patients and tissue samples

For this retrospective study, archival formalin-fixed, paraffin-embedded specimens from 230 primary NPC patients admitted from 1992-2002 to the Sun Yat-Sen University Cancer Center (Guangzhou, China) were recruited. All NPC samples were obtained before treatment with standard curative radiotherapy, with or without chemotherapy. Sixty patients were diagnosed as differentiated non-keratinized (WHO types II), and 170 patients had undifferentiated carcinoma (WHO type III). According to the Chinese 1992 staging system [13], patients were classified as 6 in stage I, 49 in stage II, 110 in stage III, 65 in stage IV. The majority of patients were male (173 of 230, or 75.2%), ranging from 86 to 14, with a median age of 46. This study was approved by the Research Ethics Committee of the Sun Yat-Sen University Cancer Center (Reference number: YP2009167).

### Tissue microarray construction

Paraffin-embedded specimens were from a previously constructed tissue microarray. Protocols and instruments for the tissue array construction were described previously [14].

### Immunohistochemistry

The immunohistochemistry (IHC) protocol was as described previously [14]. Briefly, tissue sections were de-waxed for antigen retrieval, and incubated with primary antibodies LMP1 monoclonal antibody (CS 1-4, Neomarkers, USA) at a dilution of 1:50, or p-mTOR (Ser2448), p-P70S6K (Thr389), or p-4EBP1 (Thr70) (Cell Signaling, USA) at dilutions of 1:100 overnight at 4°C. Detection was with a Catalyzed Signal Amplification Kit (DAKO Co, Carpinteria, USA) and visualization was with 3, 3'-diaminobenzidine (DAB).

IHC results were evaluated and scored independently by two pathologists without knowledge of patient clinicopathological outcomes. IHC expression levels for LMP1, p-mTOR, p-P70S6K and p-4EBP1 were assessed by a semi-quantitative scoring system according to the intensity of staining and percentage of tumor cells stained. Staining intensity was scored as 0 = negative, 1 = weak, 2 = moderate, 3 = strong. The percentage of tumor cells stained was scored as 0 = no tumor cells stained, 1 = 1-10% of tumor cells stained, 2 = 11-50% of tumor cells stained, 3 = 51-100% of tumor cells stained. The two individual parameters were added, resulting in an immunoreactivity score (IRS) ranging from 0 to 6. We defined cases with IRS ≥ 4 as high expression, and cases with IRS < 4 as low expression.

### Cell culture and plasmids

The EBV-negative human NPC cell lines HONE1 and 6-10B, and the EBV-positive NPC cell line C666-1 were incubated in RPMI-1640 medium supplemented with 10% fetal bovine serum (FBS) (Gibco, USA), 100 units of penicillin/ml and 100 μg of streptomycin/ml. All cells were maintained in a humidified incubator at 37°C with 5% CO_2_.

The eukaryotic expression plasmid pZipNeoSV-LMP1 containing the B95.8-LMP1 gene was kindly provided by Professor Kai-Tai Yao from Nan Fang Medical University (Guangzhou, China) [15].

### Transient and stable transfection

Briefly, 4 × 10^5 ^cells per well were plated into six-well plates and grown for one day in antibiotic-free medium containing 10% FBS prior to transfection. Plasmid pZipNeoSV-LMP1 and control vector transfection were performed with Lipofectamine 2000 (Invitrogen, CA) according to the manufacturer's instructions. Further assays were conducted after 48 h incubation of transiently transfected cells.

To generate the stable transfected cell lines HONE1-LMP1 and HONE1-vector, cells were passaged at 1:6 into fresh growth medium 24 h after transfection. G418 (Amresco, USA) at a final concentration of 150 μg/ml was added to complete medium to select resistant cells. Clones were separated and expanded into stable cell lines.

### Western blot analysis

Transfected cells were harvested and lysed with RIPA buffer (Upstate, USA). Denatured proteins were separated by SDS-PAGE electrophoresis and transferred to PVDF membranes (Roche, Germany), and incubated with primary antibodies LMP1 (BD, USA), p-IκBα, phosphatase and tensin homolog (PTEN), Poly ADP-ribose polymerase (PARP), Survivin, AKT1, p-AKT (Thr308) (Santa Cruz, USA), mTOR, p-mTOR(Ser2448), p-P70S6K(Thr389), p-4EBP1 (Thr70) (cell signaling, USA) and p-NF-κB p65 (Ser276) (Kangchen, China) overnight at 4°C in 5% skimmed milk/TBST (Tris-buffered saline solution containing 0.1% Tween 20) at a dilution of 1:1000. GAPDH (1:3000 dilution, Santa Cruz, USA) was used as internal control. Horseradish peroxidase-conjugated second antibody incubation was followed by chemiluminescence detection with an ECL Western blot Kit (Cell Signaling Technology, USA). Densitometry to quantify proteins was conducted by Image J 1.37 v software (NIH, USA).

### RNA extraction

Total RNA was isolated by Trizol (Invitrogen, CA) and purified by Nucleospin RNA clean-up (MN, USA). All procedures were performed according to the manufacturer's instructions. Formaldehyde agarose gel electrophoresis was carried out to quantify the total RNA.

### cDNA microarray analysis

The human 22 K oligonucleotide microarray comprised 21,329 probes from the Operon Company (Human Genome Oligo Set Version 2.1), constructed by CapitolBio Corporation (Beijing, China). Hybridization to each array was performed with equivalent amounts of HONE1-LMP1 and control HONE1-vector samples that were differentially fluorescence-labeled with Cy3 or Cy5. Fluorescence exchange experiments were performed. Hybridization and image capture were as previously described [16]. Normalization was based on a LOWESS program [17]. All original data was submitted to the Gene Expression Omnibus http://www.ncbi.nlm.nih.gov/projects/geo/ with the accession number GSM467646. Genes with signal intensity (Cy3 or Cy5) > 800 were regarded as expressed, and alteration ratios above 1.3-fold, or lower than 0.7, were defined as differential expression. Pathways analysis of all differentially expressed genes was performed according to the Kyoto Encyclopedia of Genes and Genomes (KEGG) database.

### Quantitative real-time PCR (Q-RT-PCR)

To validate the microarray results, five genes associated with the mTOR signaling pathway were analyzed by Q-RT-PCR. Primers were designed by Primer 5.0 (Additional file 1). Following the manufacturer's protocols, cDNA was prepared from 2 μg total RNA by M-MLV reverse transcriptase (Promega, USA) and amplified with a DNA Master SYBR Green I Kit (Roche, Germany). The relative expression ratio was determined by the formula 2^-ΔΔCt ^(ΔΔCt = ΔCt_HONE1-LMP1_-ΔCt_HONE1-Vector_, ΔCt = Ct_gene_-Ct_GAPDH_, where Ct is the cycle number at which the fluorescence signal exceeds background) [18].

### Small interfering RNA (siRNA) transfection

The LMP1 and negative control siRNA were chemically synthesized by GenePharma Corporation (Shanghai, China). The sequences of LMP1 siRNA (EU000388, miRNA nucleotide 371-389) were: sense sequence, 5'-GGA AUU UGC ACG GAC AGG CTT-3'; anti-sense sequence, 5'-GCC UGU CCG UGC AAA UUC CTT-3' [19,20]. The sequences of negative control siRNA were: sense sequence, 5'-UUC UCC GAA CGU GUC ACG UTT-3'; anti-sense sequence, 5'-ACG UGA CAC GUU CGG AGA ATT-3'. The EBV-positive NPC cell line C666-1 was seeded in a 24-well plate with 4×10^4 ^cells per well in growth medium without antibiotics the day before transfection. Following the manufacturer's instruction, 1 μl Lipofectamine2000 was used in each well with final siRNA concentration at 50 nM or 100 nM.

### Immunofluorescence assay

After 72 h of siRNA transfection, cells were harvested and washed thrice with PBS, suspended in PBS and centrifuged on the slides. Slides were fixed with 4% paraformaldehyde for 30 min, permeabilized, and cells covered with 0.1% Triton X-100 for 15 min. After 1 h blocking in PBS + 0.1% Tween plus 1% bovine serum albumin, cells were incubated with primary antibodies of LMP1 (BD, USA), p-mTOR (Ser2448) and p-4EBP1 (Thr70) (Cell Signaling, USA) at 4°C overnight, then with secondary antibody for 1 h at room temperature. After counterstaining with DAPI (1 μg/ml) for 10 min, slides were observed and photographed with confocal microscopy.

### Statistical analysis

Data was analyzed using SPSS16.0 software (SPSS Inc., Chicago, USA). The correlation between LMP1, p-mTOR, p-P70S6K, p-4EBP1 expression and clinicopathological parameters was assessed by chi-square test. The correlation between LMP1 and p-mTOR, p-P70S6K, p-4EBP1 expression was measured by Spearman's correlation test. Kaplan-Meier analysis and log-rank test were used to assess survival rate and compare survival rate differences. Univariate and multivariate regression analysis were performed with the Cox proportional hazards regression model to analyze the factors related to prognosis. A *p*-value less than 0.05 was considered statistically significant.

## Results

### Microarray analysis of differentially expressed genes in HONE1-LMP1 cell line

**Figure 1:**
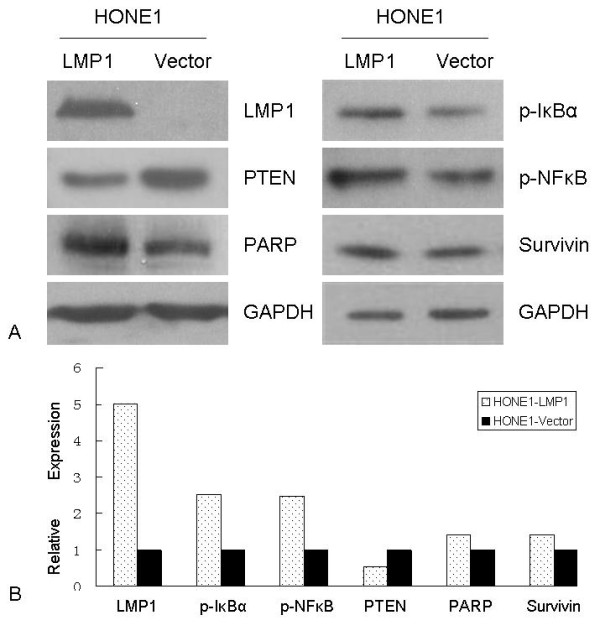
**LMP1 regulates a number of genes expressed in the NPC cell line HONE1-LMP1**. **A**. Western blot of cell lysates from HONE1-LMP1 and HONE1-vector cell lines showing that LMP1 upregulated p-IκBα, p-NF-κB, PARP and Survivin, and downregulated PTEN. GAPDH was a loading control. **B**. Changes in gene expression measured by densitometry. LMP1 increased five-fold in the HONE1-LMP1 cell line. Compared to the HONE1-vector cell line, the expression of p-IκBα and p-NF-κB increased almost 2.5-fold and a 1.4-fold increase was observed in PARP and Survivin in the HONE-LMP1 cell line. PTEN expression was reduced by half in the HONE1-LMP1 line compared to the HONE1-vector line.

As shown in Figure 1, the HONE1 cell line stably transfected with the B95.8-LMP1 plasmid showed up-regulation of NF-κB pathway downstream genes p-IκBα and p-NF-κB (2.5-fold), and PARP and survivin (1.4-fold), while down-regulation of PTEN was observed (0.5-fold).

**Figure 2:**
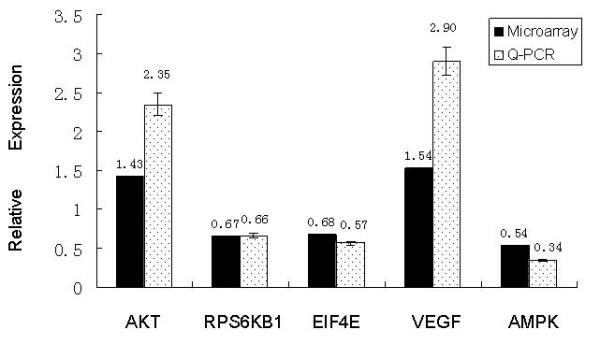
**Differentially expressed genes identified by microarray validated by Q-RT-PCR**. Five differentially expressed mTOR signaling pathway genes identified by microarray were validated by Q-RT-PCR. The numerical value above each bar is the mean alteration of the gene by microarray analysis or Q-RT-PCR. AKT and VEGF were both up-regulated, and RPS6KB1, EIF4E and AMPK were all down-regulated in the HONE1-LMP1 cell line. Q-RT-PCR analysis of these genes was normalized to GAPDH, and repeated three times independently. The mean and standard deviation are shown.

A total of 1533 genes were differentially expressed (1034 up-regulated genes and 499 down-regulated genes) in the HONE1-LMP1-transfected cells compared to those transfected with the control HONE1-Vector. Using the KEGG database, we determined that these genes clustered in several signaling pathways, including the insulin, MAPK, Wnt, TGF-beta, Notch and mTOR signaling pathways, and apoptosis. Five of the differentially expressed genes involved in the mTOR signaling pathway were validated by Q-RT-PCR (Figure 2).

### LMP1-regulated genes in mTOR signaling in NPC cell lines

**Figure 3:**
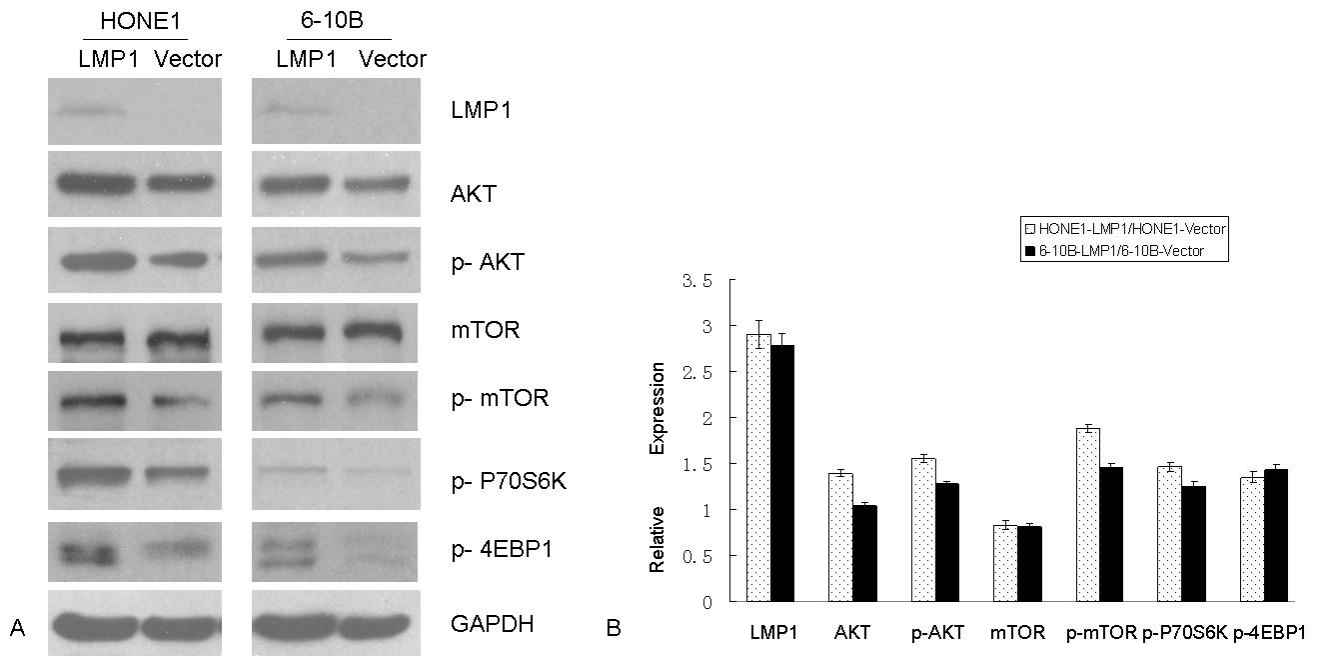
**LMP1-activates the mTOR signaling pathway through the phosphorylated AKT/mTOR/P70S6K/4EBP1 cascade in NPC cell lines**. **A**. Western blot of stable transfected NPC cell line HONE1-LMP1 and LMP1 transiently transfected NPC cell line 6-10B. Increased expression of p-AKT, p-mTOR, p-P70S6K and p-4EBP1 was seen in both the HONE1-LMP1 and 6-10B-LMP1 cell lines. GAPDH acted as a loading control. **B**. Changes in gene expression measured by densitometry. LMP1 expression increased 2.9-fold in HONE1-LMP1 cells, and 2.8-fold in 6-10B-LMP1 cells compared to the vector control. Except for expression of mTOR in both cell lines, and AKT in 6-10B-LMP1 cell line, all genes increased in expression, with the ratio ranging from 1.2 to 1.9. Experiments were repeated three times independently, and mean and standard deviation are shown.

**Figure 4:**
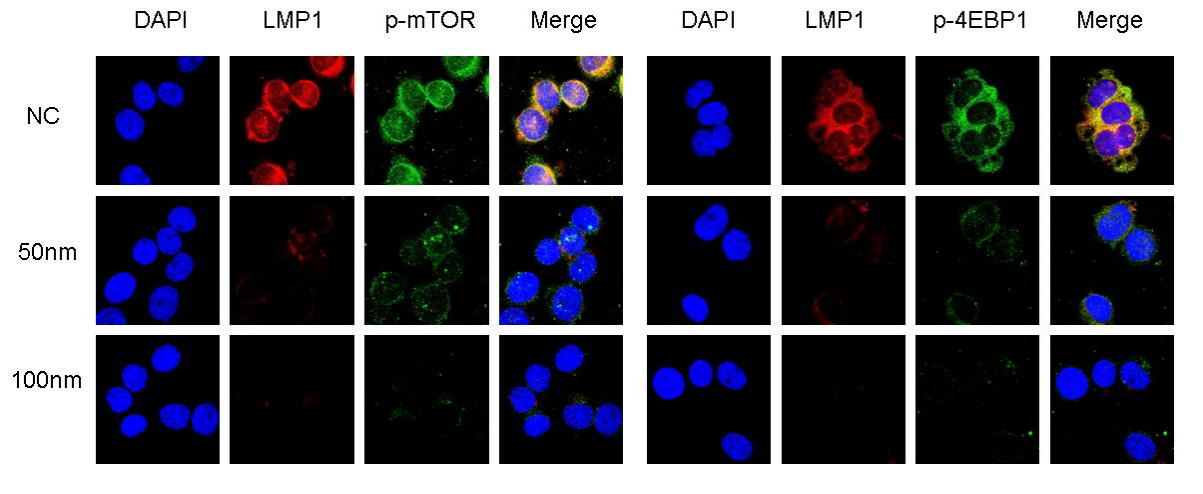
**LMP1 silencing reduced p-mTOR and p-4EBP1 expression in the C666-1 line**. C666-1 cells were harvested for immunofluoresence after 72 h LMP1-siRNA transfection at a final concentration of 50 or 100 nm. Negative control (NC)-siRNA acted as an internal standard. LMP1 expression was in the membrane and cytoplasm, with p-mTOR and p-4EBP1. The location of LMP1 and p-mTOR or p-4EBP1 in the C666-1 cell line overlapped perfectly. When LMP1 expression was decreased with 50 nm siRNA in C666-1 cells, expression of p-mTOR and p-4EBP1 was also reduced. No detectable p-mTOR or p-4EBP1 expression was observed when LMP1 was completely silenced by 100 nm siRNA in C666-1 cells.

LMP1 expression increased by 2.9-fold in HONE1 cells stably transfected with pZipNeoSV-LMP1, as measured by immunoblot. The p-AKT and p-mTOR genes, upstream in the mTOR signal pathway, were upregulated in 1.6-fold and 1.9-fold, respectively. The downstream genes p-P70S6K and p-4EBP1 were also upregulated, by 1.5-fold and 1.3-fold, respectively. When LMP1 was transiently transfected into the NPC cell line 6-10B, up-regulation of p-AKT was 1.3-fold, p-mTOR was 1.5-fold, p-P70S6K was 1.2-fold, and p-4EBP1 was 1.4-fold, consistent with results from the HONE1-LMP1 cell line (Figure 3). Immunofluorescence in the EBV-positive NPC cell line C666-1 revealed that after LMP1 knockdown with siRNA at 50 nm or 100 nm, LMP1, p-mTOR and p-4EBP1 were significantly deregulated compared to the C666-1-NC-siRNA cell line (Figure 4).

### Correlation of expression of LMP1, mTOR signaling pathway genes and clinicopathology of NPC patients

**Figure 5:**
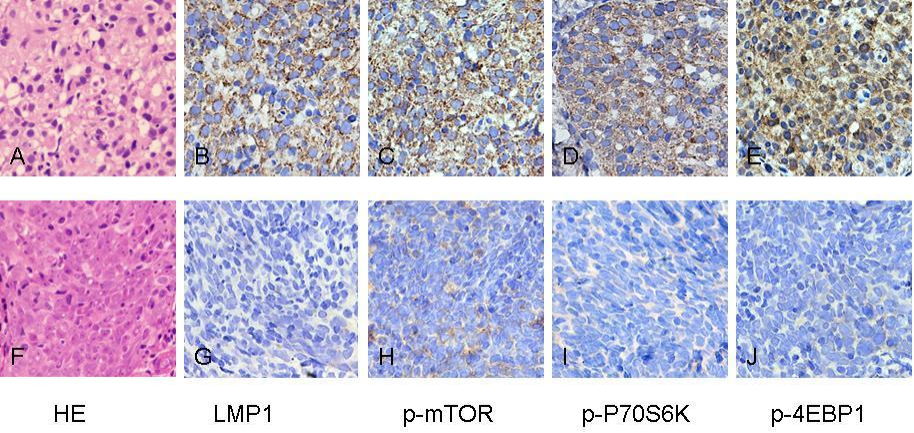
**Immunohistochemistry for LMP1, p-mTOR, p-P70S6K and p-4EBP1 in NPC biopsies**. Two representative NPC tissues are shown (200×), one in **A **to **E**, and another in frame **F **to **J**. Frames **A **and **F **are hematoxylin-eosin (H&E) staining. Positive immunostaining of LMP1 in the membrane and cytoplasm is in frame **B**, and expression of p-mTOR, p-P70S6K and p-4EBP1 with cytoplasm staining is in **C**, **D**, **E**, respectively. Samples with strong LMP1 expression also presented high levels of p-mTOR, p-P70S6K and p-4EBP1 (**B**, **C**, **D **and **E**), while samples with no LMP1 expression showed very weak or no expression of p-mTOR, p-P70S6K and p-4EBP1 (**G**, **H**, **I **and **J**).

Representative IHC staining and hematoxylin-eosin (H&E) staining of NPC tumour is shown in Figure 5. In NPC tissue with LMP1 overexpression, high levels of p-mTOR, p-P70S6K and p-4EBP1 were observed (Figure 5B-E). However, in NPC tissue with low LMP1 expression, p-mTOR, p-P70S6K and p-4EBP1 were also expressed at low levels (Figure 5G-J).

IHC staining showed membrane- and cytoplasm-positive LMP1 staining in NPC tumor cells (Figure 5B). Of the informative 224 cases, 141 (62.9%) presented with high-expression, and 83 (37.1%) presented with low LMP1 expression. Staining for p-mTOR was cytoplasmic in NPC tumor cells (Figure 5C). Of the informative 223 cases, 109 (48.9%) presented with high-expression, and 114 (51.1%) presented with low p-mTOR expression. Staining of p-P70S6K was cytoplasmic in NPC tumor cells (Figure 5D). Of the informative 224 cases, 106 (47.3%) expressed p-P70S6K at high levels, and 118 (52.7%) showed low expression. Positive staining of p-4EBP1 was seen mainly in the cytoplasm of NPC tumor cells (Figure 5E). Of the informative 223 cases, 128 (57.4%) presented with high-expression, and 95 (42.6%) of NPC presented with low expression of p-4EBP1.

A significant correlation was found between high p-mTOR expression and lymph node metastasis (*p *= 0.004) and recurrence (*p *= 0.021). High expression of p-P70S6K showed a positive correlation with distant metastasis (*p *= 0.033). High expression of p-4EBP1 correlated with lymph node metastasis (*p *= 0.045). No significant correlation was observed between LMP1 expression and gender, age, WHO type, clinical stage, recurrence, or distant metastasis (Additional file 2). Spearman's correlation analysis revealed that in NPC tumors, LMP1 expression positively correlated with expression of p-mTOR (*r *= 0.359, *p *< 0.001), p-P70S6K (*r *= 0.293, *p *< 0.001), and p-4EBP1 (*r *= 0.290, *p *< 0.001) (Table 1).

**Table 1:** Correlation between LMP1 and mTOR signaling pathway genes in NPC.

		LMP1 expression				
					
		Low	High	Case	r	*P*-value
p-mTOR expression	Low	60	54	114		
	High	19	86	105	0.359	< 0.001
				n = 219		
p-P70S6K expression	Low	57	58	115		
	High	22	81	103	0.293	< 0.001
				n = 218		
p-4EBP1 expression	Low	49	44	93		
	High	31	96	127	0.290	< 0.001
				n = 220		

### Correlation between LMP1 and mTOR expression and NPC prognosis

**Figure 6:**
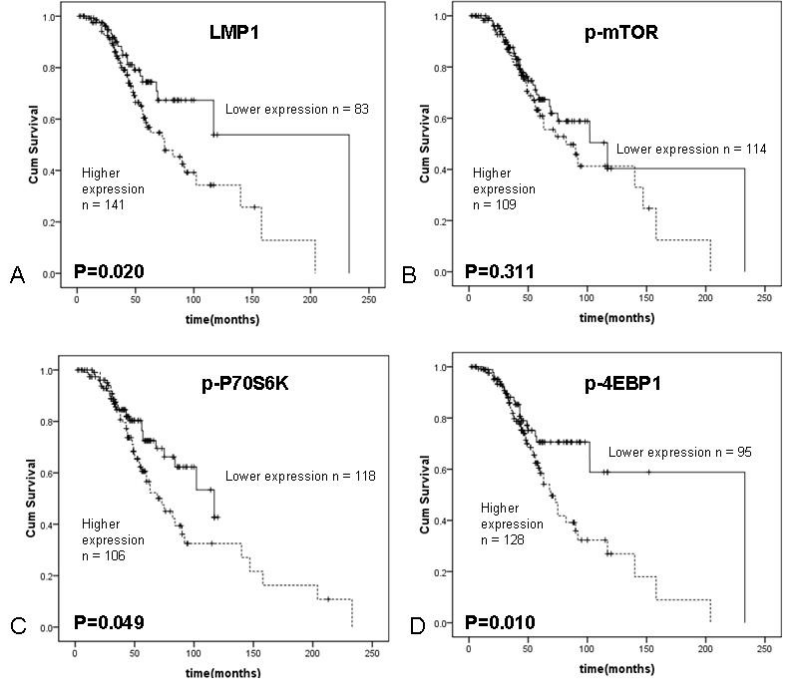
**Kaplan-Meier curves of overall NPC patient survival**. **A**, Five-year overall survival rates were 54% for patients whose NPC tumors showed high levels of LMP1 (n = 141), and 68% in patients with low LMP1 (n = 83). A significant difference was seen in overall survival rate between the groups (*p *= 0.020). **B**, Five-year overall survival rates were 55% in patients with NPC tumors with high p-mTOR expression (n = 109), and 62% in patients with low p-mTOR (n = 114). No significant difference was observed between the groups (*p *= 0.311). **C**, Five-year overall survival rates were 49% for patients with NPC tumors with high p-P70S6K expression (n = 106), and 69% for patients with low p-P70S6K expression (n = 118). A significant difference was observed between groups (*p *= 0.049). **D**, Five-year overall survival rates were 49% for patients whose NPC tumors showed high p-4EBP1 expression (n = 128), and 71% for patients with low levels of p-4EBP1 (n = 95). A significant difference was seen in the overall survival rate between the two groups (*p *= 0.010).

The overall 5-year-survival rate of the 230 NPC patients was 60%, and the 10-year-survival rate was 38%. When the patient cohort was stratified by LMP1 expression, the 5-year overall survival rate in patients with high LMP1 expression (n = 141) was 54%, and with low LMP1 expression (n = 83), it was 68%. The two groups showed a significant difference (*p *= 0.020, Figure 6A). For p-mTOR expression, the 5-year overall survival rates in NPC patients with high expression (n = 109) was 55%, and was 62% for patients with low expression (n = 114), with no significant difference between the two groups (*p *= 0.311, Figure 6B). For p-P70S6K expression, the 5-year overall survival rate for NPC patients with high expression (n = 106) was 49%, and for low expression (n = 118) it was 69%, with a significant difference between the two groups (*p *= 0.049, Figure 6C). For p-4EBP1, the 5-year overall survival rates in patients with high expression (n = 128) was 49%, and for low expression (n = 95) it was 71%, with a significant difference between the groups (*p *= 0.010, Figure 6D).

Univariate analysis showed gender, age, clinical stage, metastasis, LMP1 expression and p-4EBP1 expression were prognostic predictors of overall survival in NPC patients (Table 2). Multivariate Cox regression analysis indicated that high expression of LMP1, gender and metastasis, were independent prognostic factors in the NPC patients, but mTOR signaling pathway genes were not (Table 2).

**Table 2:** Univariate and multivariate analysis of Cox proportional hazards model in NPC.

Characristics	Hazard ratio	95% CI	P-value
**Univariate analysis**			
Gender (male vs. female)	0.422	0.232-0.770	0.005*
Age (< 46 vs. ≥ 46)	1.721	1.085-2.729	0.021*
WHO type (I vs. IV)	1.025	0.618-1.700	0.925
Clinical stage (I + II vs. III + IV)	1.824	1.012-3.286	0.045*
T stage (T1~2 vs. T3~4)	1.571	0.972-2.540	0.065
N stage (N0 vs. N1~3)	0.936	0.568-1.544	0.796
Recurrence (no vs. yes)	1.442	0.895-2.322	0.132
Metastasis (no vs. yes)	2.466	1.412-4.304	0.002*
LMP1 expression (low vs. high)	1.863	1.091-3.183	0.023*
p-mTOR expression (low vs. high)	1.274	0.796-2.040	0.314
p-P70S6K expression (low vs. high)	1.447	0.907-2.309	0.121
p-4EBP1 expression (low vs. high)	1.920	1.154-3.195	0.012*
**Multivariate analysis**			
Gender (male vs. female)	0.447	0.234-0.851	0.014*
Metastasis (no vs. yes)	2.591	1.383-4.856	0.003*
LMP1 expression (low vs. high)	2.056	1.161-3.641	0.013*

* Statistically significant difference.

## Discussion

Previous studies reported that LMP1 is involved in several signaling pathways including NF-κB, AP-1, JAK/STAT, PI3K/AKT and ERK-MAPK and regulate their downstream effects [6-9]. LMP1 activate the PI3K/AKT/mTOR signaling pathway in B lymphocytes [21], and the mTOR signaling pathway has been identified as a downstream component of the PI3K/AKT pathway in the LMP2A-transfected NPC cell lines HONE1 and AD/AH [22]. The mTOR signaling pathway might positively regulate cyclin D1 expression in NPC [23]. In this study, microarray analysis of the NPC HONE1 cell line stably transfected with LMP1 identified several differentially expressed genes of mTOR signaling pathways. This is the first report that LMP1 can regulate the mTOR signaling pathway in NPC. Furthermore, LMP1 overexpression and knockdown studies confirmed that LMP1-regulated genes are involved in the mTOR signaling pathway, and LMP1 expression was essential for the activation of p-mTOR and p-4EBP1 in NPC cell lines. In addition, our *in vitro *studies found that LMP1 expression positively correlated with overexpression of p-mTOR, p-P70S6K and p-4EBP1 in NPC tumors.

As a well-known oncogene, one of the functions of LMP1 is to promote cell proliferation in NPC [24,25]. The mTOR signaling pathway is also a major effector in cell growth, cell proliferation and cell survival, through regulation of protein synthesis, while P70S6K and 4EBP1 play particularly important roles in the mTOR signaling pathway growth acceleration function [10]. In this study, our findings suggest that activation of P70S6K and 4EBP1 requires LMP1, and that when these genes are phosphorylated by LMP1, activated P70S6K and 4EBP1 initiate a sequence of events that promotes protein synthesis, cell growth and proliferation. Further studies need to be done to investigate the mechanism by which LMP1 regulates mTOR signaling in NPC tumorigenesis.

Deregulation of the mTOR signaling pathway is reported in many malignancies, and some of the signaling molecules in this pathway are predictors of prognosis in different types of cancers. Cytoplasmic p-mTOR expression correlates with poorer survival in gastric cancer and cervix adenocarcinoma [26,27]. High expression of p-mTOR, p-P70S6K and p-4EBP1 correlate with poor outcome in glioblastoma [28], and p-4EBP1 was demonstrated to be a potential prognostic factor in breast cancer and an independent prognostic marker in ovarian cancer [29,30]. Our results revealed that NPC patients with high p-P70S6K and p-4EBP1 expression had a significantly shorter overall survival than those with low p-P70S6K (*p *= 0.049) and p-4EBP1 (*p *= 0.010) expression. These results are in accordance with previous studies on malignancies. p-P70S6K is required for 5'-TOP mRNA translation, especially translation of all ribosomal proteins, elongation factors, and poly (A)-binding protein. 4EBP1 forms a complex with eIF4E by closely interaction, and once 4EBP1 is phosphorylated, 4EBP1 loses its high affinity for eIF4E. When eIF4E dissociates, activated 4EBP1 enhances protein synthesis [11,12]. High expression of p-P70S6K and p-4EBP1 in NPC tissues might result in a high level of protein synthesis and cell proliferation, and the poor prognosis of the NPC patients.

In this study, a large sample size of NPC cases were used for IHC staining of LMP1, and LMP1 overexpression was detected in 62.9% (141/224) of NPC tumors, in accordance with previous studies [31-33]. Interestingly, we found that LMP1 overexpression in NPC patients was significantly associated with poorer overall survival (*p *= 0.020). This result differed from previous reports, which found that LMP1 overexpression suggested a better prognosis of NPC patients [34], and LMP1 was not an effective indicator of NPC outcomes [35]. The possible reasons for the differences might be different sample sizes, regional distribution, or different LMP1 variants. Compared to previous studies, our study had a larger sample size for LMP1 expression and NPC prognosis.

Although high-expression of LMP1, p-P70S6K and p-4EBP1 was associated with poor survival of NPC patients, multivariate analysis revealed that only LMP1 expression (*p *= 0.013), as well as gender (*p *= 0.014) and metastasis (*p *= 0.003), were independent prognostic factors. We found that the mTOR signaling pathway was triggered by LMP1, suggesting that LMP1 may have more important roles than mTOR signaling molecules in the carcinogenesis and development of NPC.

## Conclusions

In summary, we present the first report that LMP1-regulated genes are involved in the mTOR signaling pathway, and LMP1 expression is essential for the activation of the mTOR signaling pathway in NPC. LMP1 activates the AKT/mTOR/P70S6K/4EBP1 axis in NPC tumors, and high expression of LMP1, p-P70S6K and p-4EBP1 predict poor prognosis of NPC patients.

## Competing interests

The authors declare that they have no competing interests.

## Authors' contributions

JC carried out substantial experimental work and drafted the manuscript. JYS designed the research and supervised the study. CFH was responsible for the patient samples and tissue array construction. JHH and QS supported the work of IHC. LXY supported the lab work. XFZ and YXZ helped finalize the research design. All authors read and approved the final manuscript.

## Supplementary Material

Additional file 1 **Table for primers used in the study**. The table shows the primers of five genes associated with the mTOR signaling pathway which were designed by Primer 5.0. Click here for file

Additional file 2 **Correlation between LMP1, p-mTOR, p-P70S6K, p-4EBP1 and clinicopathological parameters of NPC**. The table shows the correlation between the expression of LMP1, p-mTOR, p-P70S6K, p-4EBP1 and clinicopathological parameters of NPC (including gender, age, WHO type, TNM stage, T stage, N stage, recurrence and metastasis). Click here for file
