# Supplementary material for: Epstein-Barr virus encoded latent membrane protein 1 regulates mTOR signaling pathway genes which predict poor prognosis of nasopharyngeal carcinoma
Source: J Transl Med. 2010 Mar 26;8:30. doi: 10.1186/1479-5876-8-30 (PMC2861642; doi:10.1186/1479-5876-8-30)
Supplement: Additional file 1 — Table for primers used in the study. The table shows the primers of five genes associated with the mTOR signaling pathway which were designed by Primer 5.0. [file 1479-5876-8-30-S1.DOC]

Additional file 1: PCR primers used in the study

| Gene | GenBank acession number | Primer sequence | Anneal temperature | Product length |
| --- | --- | --- | --- | --- |
| AKT | NM_005163 | Forward 5'-ACGTCGGAGACTGACACCA-3' | 59℃ | 192bp |
|  |  | Reverse 5'-CCTCCAAGCTATCGTCCAG-3' |  |  |
| RPS6KB1 | NM_003161 | Forward 5'-TAAAGCATCCCTTCATCG-3' | 59℃ | 139bp |
|  |  | Reverse 5'-CAGGCAGTGTCTTCCATA-3' |  |  |
| EIF4E | NM_001968 | Forward 5'-ACGGAATCTAATCAGGAGGTT-3' | 55℃ | 247bp |
|  |  | Reverse 5'-CTTCCCACATAGGCTCAATAC-3' |  |  |
| VEGF | NM_001033756 | Forward 5'-GGCTGCTGCAATGACGAG-3' | 59℃ | 184bp |
|  |  | Reverse 5'-AAGGCCCACAGGGATTTT-3' |  |  |
| AMPK | NM_206907 | Forward 5'-AAACAGGCTCCACGAAGG-3' | 57℃ | 138bp |
|  |  | Reverse 5'-GTGCATCAAGCAGGACATT-3' |  |  |
| GAPDH | NM_002046 | Forward 5'-CCACCCATGGCAAATTCCATGGCA-3' | 58℃ | 588bp |
|  |  | Reverse 5'-TCTAGACGGCAGGTCAGGTCCACC-3' |  |  |
