# Supplementary material for: Epstein-Barr virus encoded latent membrane protein 1 regulates mTOR signaling pathway genes which predict poor prognosis of nasopharyngeal carcinoma
Source: J Transl Med. 2010 Mar 26;8:30. doi: 10.1186/1479-5876-8-30 (PMC2861642; doi:10.1186/1479-5876-8-30)
Supplement: Additional file 2 — Correlation between LMP1, p-mTOR, p-P70S6K, p-4EBP1 and clinicopathological parameters of NPC. The table shows the correlation between the expression of LMP1, p-mTOR, p-P70S6K, p-4EBP1 and clinicopathological parameters of NPC (including gender, age, WHO type, TNM stage, T stage, N stage, recurrence and metastasis). [file 1479-5876-8-30-S2.DOC]

**Additional file 2**

**Title: Correlation between LMP1, p-mTOR, p-P70S6K, p-4EBP1and clinicopathological parameters of NPC**

| Variable | | Case | LMP1 expression | | *P*-value | Case | p-mTOR expression | | *P*-value | Case | p-P70S6K expression | | *P*-value | Case | p-4EBP1 expression | | *P*-value |
| --- | --- | --- | --- | --- | --- | --- | --- | --- | --- | --- | --- | --- | --- | --- | --- | --- | --- |
|  |  |  | Low | High |  |  | Low | High |  |  | Low | High |  |  | Low | High |  |
|  |  | n=224 | n=83 | n=141 |  | n=223 | n=114 | n=109 |  | n=224 | n=118 | n=106 |  | n=223 | n=95 | n=128 |  |
| Gender | Male | 169 | 57 | 112 | 0.079 | 169 | 89 | 80 | 0.438 | 170 | 89 | 81 | 0.877 | 170 | 70 | 100 | 0.525 |
|  | Female | 55 | 26 | 29 |  | 54 | 25 | 29 |  | 54 | 29 | 25 |  | 53 | 25 | 28 |  |
| Age | ＜46 | 105 | 42 | 63 | 0.409 | 105 | 58 | 47 | 0.284 | 105 | 60 | 45 | 0.229 | 103 | 51 | 52 | 0.058 |
|  | ≥46 | 119 | 41 | 78 |  | 118 | 56 | 62 |  | 119 | 58 | 61 |  | 120 | 44 | 76 |  |
| WHO type | Ⅱ | 58 | 19 | 39 | 0.528 | 58 | 29 | 29 | 0.879 | 58 | 30 | 28 | 0.880 | 58 | 23 | 35 | 0.645 |
|  | Ⅲ | 166 | 64 | 102 |  | 165 | 85 | 80 |  | 166 | 88 | 78 |  | 165 | 72 | 93 |  |
| TNM stage | Ⅰ+Ⅱ | 55 | 21 | 34 | 0.873 | 52 | 25 | 27 | 0.638 | 51 | 26 | 25 | 0.873 | 53 | 24 | 29 | 0.751 |
|  | Ⅲ+Ⅳ | 169 | 62 | 107 |  | 171 | 89 | 82 |  | 173 | 92 | 81 |  | 170 | 71 | 99 |  |
| T stage | T1～2 | 93 | 38 | 55 | 0.330 | 89 | 47 | 42 | 0.684 | 89 | 47 | 42 | 1.000 | 90 | 43 | 47 | 0.216 |
|  | T3～4 | 131 | 45 | 86 |  | 134 | 67 | 67 |  | 135 | 71 | 64 |  | 133 | 52 | 81 |  |
| N stage | N0 | 58 | 18 | 40 | 0.343 | 58 | 20 | 38 | 0.004* | 59 | 25 | 34 | 0.070 | 58 | 18 | 40 | 0.045* |
|  | N1～3 | 166 | 65 | 101 |  | 165 | 94 | 71 |  | 165 | 93 | 72 |  | 165 | 77 | 88 |  |
| Recurrence | No | 168 | 65 | 103 | 0.427 | 167 | 93 | 74 | 0.021* | 165 | 92 | 73 | 0.131 | 165 | 73 | 92 | 0.443 |
|  | Yes | 56 | 18 | 38 |  | 56 | 21 | 35 |  | 59 | 26 | 33 |  | 58 | 22 | 36 |  |
| Metastasis | No | 193 | 67 | 126 | 0.075 | 194 | 97 | 97 | 0.430 | 193 | 96 | 97 | 0.033* | 193 | 81 | 112 | 0.693 |
|  | Yes | 31 | 16 | 15 |  | 29 | 17 | 12 |  | 31 | 22 | 9 |  | 30 | 14 | 16 |  |

The cases with IRS ≥ 4 as high expression, cases with IRS ＜ 4 as low expression. * Statistically significant difference.
